# Strengthening of RC Beams Using Externally Bonded Reinforcement Combined with Near-Surface Mounted Technique

**DOI:** 10.3390/polym8070261

**Published:** 2016-07-19

**Authors:** Kh Mahfuz ud Darain, Mohd Zamin Jumaat, Ahmad Azim Shukri, M. Obaydullah, Md. Nazmul Huda, Md. Akter Hosen, Nusrat Hoque

**Affiliations:** 1 Centre for Innovative Construction Technology (CICT), Department of Civil Engineering, Faculty of Engineering, University of Malaya, 50603 Kuala Lumpur, Malaysia; ahmadazimshukri@gmail.com (A.A.S.); md_obaydullah@yahoo.com (M.O.); nazmulhuda.128@gmail.com (M.N.H.); enggakter@gmail.com (M.A.H.); nusrat_hoque@yahoo.com (N.H.); 2 Architecture Discipline, Science, Engineering and Technology School, Khulna University, 9208 Khulna, Bangladesh

**Keywords:** CEBNSM, CFRP, externally bonded, near surface mounted, moment-rotation analysis

## Abstract

This study investigates the flexural behaviour of reinforced concrete (RC) beams strengthened through the combined externally bonded and near-surface mounted (CEBNSM) technique. The externally bonded reinforcement (EBR) and near-surface mounted (NSM) techniques are popular strengthening solutions, although these methods often demonstrate premature debonding failure. The proposed CEBNSM technique increases the bond area of the concrete–carbon fibre reinforced polymer (CFRP) interface, which can delay the debonding failure. This technique is appropriate when any structure has a narrow cross-sectional width or is in need of additional flexural capacity that an individual technique or material cannot attain. An experimental test matrix was designed with one control and five strengthened RC beams to verify the performance of the proposed technique. The strengthening materials were CFRP bar as NSM reinforcement combined with CFRP fabric as EBR material. The test variables were the diameter of the NSM bars (8 and 10 mm), the thickness of the CFRP fabrics (one and two layers) and the U-wrap anchorage. The strengthened beams showed enhancement of ultimate load capacity, stiffness, cracking behaviour, and strain compatibility. The ultimate capacity of the CEBNSM-strengthened beams increased from 71% to 105% compared to that of the control beam. A simulation method based on the moment-rotation approach was also presented to predict the behaviour of CEBNSM-strengthened RC beams.

## 1. Introduction

Throughout the world, the growing interest in the sustainability of construction encourages the engineering community to develop policies that discourage new construction rather than extend the design life of existing structures [1]. Structural strengthening allows existing underperforming structures to survive against additional service load requirement, design, or construction error and structural deterioration due to age or the surrounding environment [2,3,4]. In the past decade, fibre-reinforced polymer (FRP) has substituted conventional strengthening materials such as steel and concrete because of its high strength-to-weight ratio, resistance to corrosion, and low density [5,6,7,8,9,10]. The externally bonded reinforcement (EBR) and near-surface mounted (NSM) strengthening techniques are gaining popularity. The EBR technique consists of one or multiple FRP laminates that are bonded on the tension side of the strengthened member [11]. Meanwhile, the NSM technique involves the insertion of FRP strips or rods into pre-cut grooves in concrete covers and then filling up with epoxy adhesives [12]. The NSM technique is a contemporary technique that offers a high level of strengthening efficacy, is less prone to premature debonding failure, and enhances protection against fire, mechanical damage, the effects of aging, and acts of vandalism. The technique also demonstrates better durability, stress-sharing mechanisms, and fatigue performance, given that the reinforcement is located inside [13].

The problem faced with the EBR method is normally in the form of premature debonding due to high interfacial shear stresses between the FRP and the concrete substrate at the sheet of FRP curtailment location [14,15,16]. The thickness of FRP composite plays an important role regarding this issue, where the reduction of plate thickness drives down the magnitude of stress concentration at the plate ends [17]. For a fixed FRP ratio, the debonding potential has been reported to increase significantly with increasing FRP thickness [18]. Oehlers [19] proposed a formula based on the interaction between flexural and shear capacities of the beam where the de-bonding failure moment is inversely proportional to FRP sheet thickness.

In the NSM method, often, the width of the beam may not be wide enough to provide necessary edge clearance and clear spacing between two adjacent NSM grooves. The American Concrete Institute (ACI) proposed a minimum edge clearance and clear spacing between two adjacent NSM grooves supported by the research of De Lorenzis, Blaschko, and Parretti, and Nanni [20,21,22]. This strengthening technique necessitates more concrete cover to allocate enough space for cutting grooves without any possibility of damaging the steel. However, lots of existing structures have less concrete cover due to faulty construction or for a number other reasons, posing a major challenge to this technique [23]. Recently, for retrofitting of reinforced concrete (RC) members with deteriorated cover concrete, the effectiveness of a new strengthening technique (Inhibiting-Repairing-Strengthening, IRS) was experimentally evaluated [24,25,26]. It consists of the installation of an innovative composite system—made of inorganic matrix and stainless steel strip/fabric—in the thickness of the cover concrete during the repairing/restoring of the same.

With regard to these limitations, a hybrid strengthening method between the EB and NSM method is proposed. The strengthening method—which will be called the combined externally bonded and near-surface mounted (CEBNSM) technique—offers a prudent and optimum combination of NSM and EBR techniques, which perform to complement each other and get rid of their limitations reciprocally. Previous work on similar hybrid strengthening involved a hybrid between NSM steel bars and EB steel plates, as introduced by Rahman, et al. [27]. The use of steel instead of FRP was proposed by Rahman, et al. [27] due to the higher ductility of steel; however, this increase in ductility was not very prominent, as all of the strengthened beams prematurely failed by concrete cover separation.

This study proposes the development of another type of CEBNSM strengthening technique involving the use of EB FRP sheets with NSM FRP bars or NSM steel bars, with the aim of developing a cost-effective strengthening solution which will delay or avoid the debonding failure seen in the previous study. It is identified that the drop in Carbon Fiber Reinforced Polymer (CFRP) fabric thickness diminishes the degree of stress concentration at the fabric edge [27,28]. Through combination, it is possible to reduce the CFRP fabric thickness by transferring a part of the required total strengthening area of CFRP fabric material from EBR to NSM technique. Consequently, the NSM bar or strip size can also be reduced through sharing with EBR strengthening material, and thus provide sufficient space for edge clearance and groove clear spacing [29,30], which can help reduce the possibility of concrete cover separation failure. Moreover, the NSM groove itself creates more contact surface area between the FRP composite and the concrete substrate at the cross-section. As stress is equal to the load divided by the corresponding surface area, an increase in surface area will decrease interfacial stress, further reducing the possibility of concrete cover separation. The addition of adhesive in the NSM grooves to the CEBNSM system also improves bond performance between the strengthening CFRP fabric and the concrete substrate.

The experimental results for six beams are presented in this study, where five of the beams are strengthened with CEBNSM technique. The experimental load, deflection, crack spacing, crack width and strain values of the strengthened beams were analysed to evaluate the serviceability behaviour, ductility, and flexural performance of the proposed CEBNSM technique. A simulation method based on the moment-rotation approach was also presented to predict the behaviour of the CEBNSM-strengthened reinforced concrete beam.

## 2. Materials and Methods

### 2.1. Test Matrix

The experimental program was designed with six RC beams. Of those beams, one was assigned as the control specimen, and the remaining beams were strengthened with the CEBNSM strengthening technique. The main testing variables were the diameter of the slotted reinforcement (8 and 10 mm), the thickness of the external CFRP fabrics (one and two layers), and the anchorage at the curtailment location. The length of the CFRP fabric was also varied. For the single-ply condition, a 2900 mm-long CFRP fabric was bonded at the beam soffit. However, for the double-ply condition, the second layer was 2600 mm to avoid end peeling failure due to the increased normal stress developed at the curtailment end of the CFRP fabric [10,31]. The detailed test matrix is shown in Table 1. The beam notation is explained as follows, using “CBC10P2A” as an example. Specifically, C denotes the combination technique, BC denotes the bar as CFRP NSM reinforcement, 8 and 10 denotes the 8 or 10 mm diameter NSM bar, P1 and P2 denote the single-ply or double-ply of CFRP fabric through the EBR technique, and A denotes the anchorage.

### 2.2. Specimens and Materials

**Figure 1:**
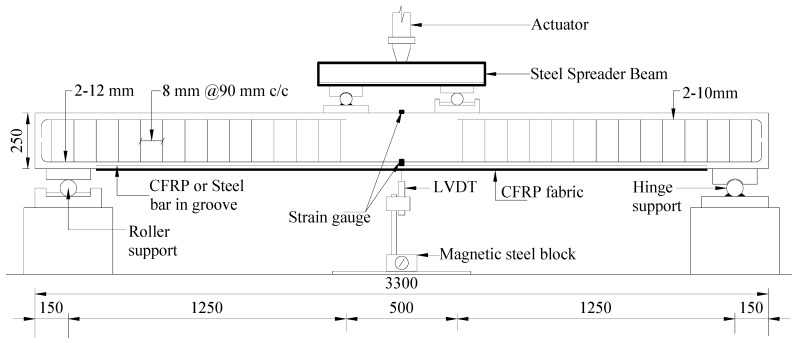
Beam details and test setup (all dimensions are in mm). CFRP: carbon fibre reinforced polymer; LVDT: linear variable differential transducer.

In this experimental program, the dimension of the rectangular RC beams was 3300 mm × 250 mm × 125 mm with a clear span of 3.0 m (Figure 1). The steel ratio (ρ = *As*/*bd*) was 0.0085 to constitute an under-reinforced RC beam. Two 12 and 10 mm diameter deformed bars were used as the bottom and top reinforcements, respectively, having an 8 mm diameter stirrup with a 90 mm spacing. The top reinforcement and the shear reinforcement were discontinued. They were avoided in the maximum moment region to assure flexural failure.

Ready-mix concrete was used for concrete casting. During casting, the same concrete batch was used for the six beams to maintain the same concrete properties. The coarse and fine aggregates were from crushed stone and quarry sand, respectively. The mechanical properties of concrete—such as compressive and flexural strengths—were evaluated 28 days after concrete casting, based on the cube (100 mm × 100 mm × 100 mm) and prism (500 mm × 100 mm × 100 mm) specimens, according to [32,33]. The mechanical properties of concrete are listed in Table 2.

The mechanical properties of the steel bar were checked in the laboratory following the [34] guidelines to confirm the specifications given by the suppliers. Either 8 mm or 10 mm diameter CFRP strengthening bar—which was spirally wound with a fibre tow—was placed at the groove, which had a density of 1.65 g/mm^2^. A unidirectional, woven CFRP fabric (SikaWrap^®^-301C) with a thickness of 0.17 mm was used as external strengthening material at the beam soffit. A two-part epoxy (Sikadur^®^ 30) was used as the adhesive filler to fix the CFRP bar inside the groove. The CFRP fabric was also soaked with epoxy resin (Sikadur^®^ 330) for proper bonding with the concrete substrate. These resins are available with a two-part adhesive based on a combination of epoxy resins and filler. Table 2 presents the properties of concrete, internal steel, strengthening steel, CFRP bar, CFRP fabric, and epoxy.

### 2.3. Specimen Design and Preparation

**Figure 2:**
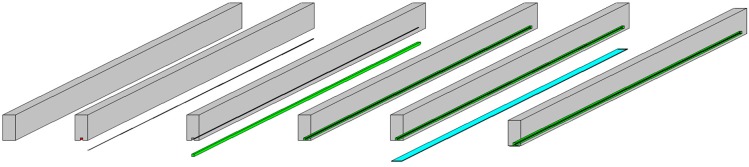
Sequence of specimen preparation and strengthening. Epoxy and CFRP fabric are colored green and light blue respectively.

The specimen preparation and strengthening processes are illustrated in Figure 2. A superior quality diamond-bladed concrete saw was used to create 16 mm × 16 mm and 24 mm × 24 mm grooves at the tension region of the RC beam to accommodate the 8 and 10 mm-diameter CFRP bars. The groove dimension of the bar diameter was set as two times the diameter of the CFRP bar, as suggested by [23] for the optimal groove size. After using the cutter, the concrete lugs were manually taken out with a hand chisel and a hammer. The remaining rough concrete surface was cleaned using a high-pressure air jet. Acetone was applied on the surface to remove any fine dust particle and any oily substance present in the groove to make a good bonding surface for strengthening. Masking tape was affixed along the ridge of the groove line to ensure the proper placement of epoxy inside the groove and neat finishing of the epoxy surface. Then, approximately two-thirds of the groove was filled with epoxy (Sikadur^®^ 30), which was prepared according to the directions of the manufacturers. The NSM CFRP bar was properly cleaned and gently pressed inside the groove until surrounded by an equal amount of epoxy. Then, a spatula was used to level the surface and clean the area. The whole preparation was left for standard curing time, as prescribed by the manufacturer.

After the curing period, the remaining cement laitance and loose materials were removed from the concrete surface with the help of an abrader to ensure superior bonding of the concrete–CFRP assemblage. Then, the surface was cleaned with a brush and a high-pressure air jet. Finally, acetone was used on the concrete surface before the wet layup process. Following the instructions of the manufacturers, a layer of epoxy was spread on the surface and then the CFRP fabric was laid over it. A recommended roller was pressed firmly on the fabric layer until the adhesive was squeezed out through the tiny pores of the CFRP fibre. Before the test, the sample was left for standard curing time.

Using suitable support conditions, the test was conducted using a 500 kN load-carrying capacity Instron universal testing machine under a four-point bending load (Figure 1). Deflection was measured using the linear variable differential transducer (LVDT), which was placed at the centre of the maximum moment region. The 5 mm strain gauges were affixed at the centre of the internal steel bars. For measurement of the strain value of the strengthened CFRP and steel bars, the 5 mm-long strain gauges were planted at the central point, which were 500 and 1250 mm away from the centre of the strengthening bar. In the case of the CFRP fabric, 30 mm-long special strain gauges were installed at the central point, which was 250, 500, 1,250, and 1400 mm away from the centre point of the CFRP fabric. The 30 mm-long strain gauges were positioned at the uppermost surface of the concrete beam to measure concrete compressive strain. Transverse strains along the mid-span depth of the beams were measured using Demec points. Micro-cracks along the side of the concrete surface were measured using a DinoLite digital microscope.

## 3. Results and Discussion

The experimental results of the CEBNSM-strengthened RC beams are arranged in Table 3. These beams were strengthened with the CFRP bar inside the NSM groove and the CFRP fabric bonded at the beam soffit. The main test variables were the bar diameter (8 and 10 mm), the thickness of the CFRP fabric layer (one and two layer), the anchorage (with and without) at the cut-off zone of the EBR CFRP fabric layer (one and two layer), and the anchorage (with and without) at the cut-off point of the EBR CFRP fabric. Results are expressed in terms of their first crack load-carrying capacity, yield load-carrying capacity, and ultimate load-carrying capacity.

### 3.1. Load-Carrying Capacity

Table 3 provides the results obtained from the experimental tests carried out on one control beam and five CEBNSM-strengthened RC beams. The addition of the strengthening material to the RC beams caused superior load-carrying capacity, reduced ultimate deflection, and reduced the possibility of the debonding problem. The ultimate load-carrying capacity increased by 82%, 97%, 110%, 124%, and 170% for the CBC8P1-, CBC8P2-, CBC10P1-, CBC10P2-, and CBC10P2A-strengthened beams, respectively, compared with the control beam. The corresponding first crack load-carrying capacity and yield load-carrying capacity of the beams significantly improved after strengthening. The yield point was determined by the stiffness variation in the load–deflection curve, as well as the internal steel yielding point from the corresponding load–steel strain diagram. The average increment of the ultimate load-carrying capacity was 116.5%, compared to that of the control beam. This enhanced ultimate load-carrying capacity shows the superior performance of the strengthened beams compared with that of the control beam.

**Figure 3:**
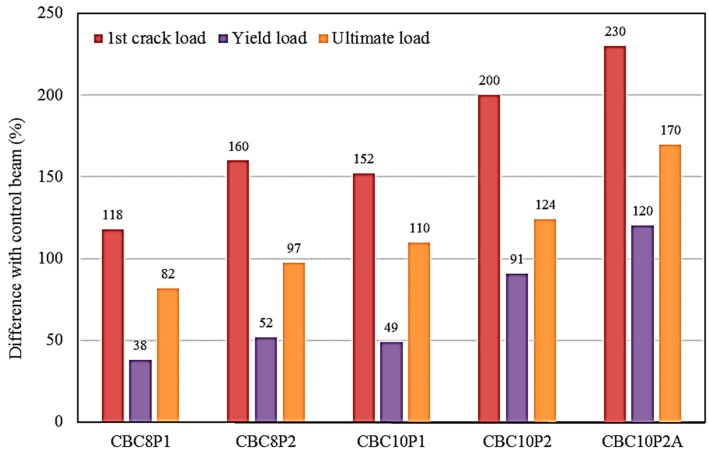
Percentile increment of the first crack, yield, and ultimate load-carrying capacities of combined externally bonded and near-surface mounted (CEBNSM)-strengthened beams compared with the control beam.

The percentile increment of the first crack load-carrying capacity, yield load-carrying capacity, and ultimate load-carrying capacity are illustrated in Figure 3. The first crack load-carrying capacity, yield load-carrying capacity, and ultimate load-carrying capacity were significantly improved by the CEBNSM technique. Among these three load states, the first crack load-carrying capacity was significantly improved for all CEBNSM-strengthened beams. The range of the first crack load-carrying capacity improvement was 118% to 230% compared with that of the control beam. This serviceability improvement is one of the positive features of this technique, given that the early first crack load welcomes various environmental agents, which would aggravate the cracking condition and eventually be responsible for further deterioration. The range of the yield load-carrying capacity improvement is lower than that of the first crack load and the ultimate load. This range was 38% to 120% compared with that of the control beam. Figure 3 shows that the average yield load-carrying capacity of the CEBNSM-strengthened beams was approximately 73% of their ultimate load-carrying capacity.

The average increment of the yield load-carrying capacity is 70% compared with that of the control beam, and its trend shows the lowest gain compared with the corresponding first crack load-carrying capacity and ultimate load-carrying capacity of the strengthened beams. The percentage increment of the ultimate load-carrying capacity was 82% to 170% compared with that of the control beam. Typically, beams with a high area of strengthening material exhibit an enhanced ultimate load-carrying capacity.

### 3.2. Load–Deflection Diagram

**Figure 4:**
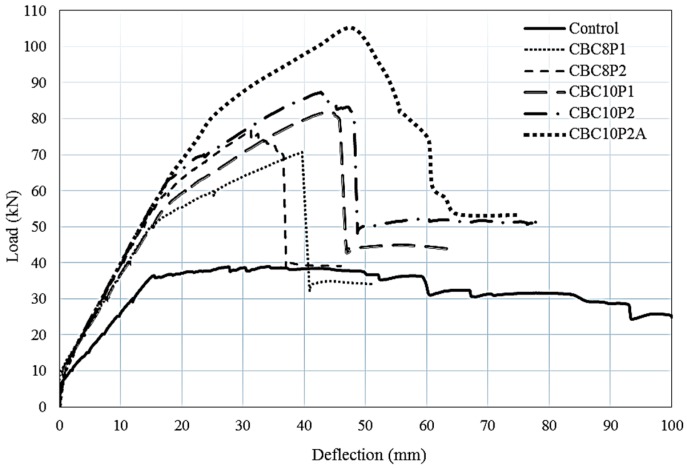
Load–deflection diagram of tested beams.

The load–deflection relationship of the control and strengthened RC beams is depicted in Figure 4. The unstrengthened RC beam showed the typical behaviour, with crack and yield point followed by a nonlinear portion at the post-yield stage. All of the CEBNSM-strengthened RC beams exhibited a trilinear response up to the ultimate load. The first fragment of the curve varied in a linear manner with minor deflection until the first crack appeared. For all the strengthened beams, this technique contributed to the increment of the first crack load.

The second segment was the post-crack to yield stage of the internal reinforcement of the beams. Strengthened beams exhibited a considerable stiffness improvement in this stage compared with the control beam. At this stage, the internal steel reinforcement and the strengthening materials exhibited the tensile stresses of the beam. The average pre-yield stiffness increment of the strengthened beam was 36% compared with the control beam. The CBC8P2-strengthened beam showed a maximum 50% more pre-yield stiffness compared to the control beam. With the prevention of the further expansion of flexural cracks, the CFRP bar contributed to the enhancement of the moment of inertia of the cracked section.

The third stage of the load–deflection graph continued from yielding up to failure. This segment exhibited better improvement in terms of strength and stiffness. In this post-yield stage, most of the tension stresses are resisted by the NSM reinforcement and the EBR CFRP fabric because of the yielding of the tension steel. The strengthened beams exhibited 83% more average pre-ultimate stiffness increment than that of the control beam. CBC8P2-strengthened beam showed a maximum post-yield stiffness of approximately 116% more than that of the control beam. The anchored CBC10P2A-strengthened beams exhibited 93% more stiffness increment than that of the control beam. The post-peak response of the load–deflection curve of strengthened beams was characterized by a sudden drop in load carrying capacity, regardless of the FRP fracture (flexural failure) or premature (debonding) failure.

**Figure 5:**
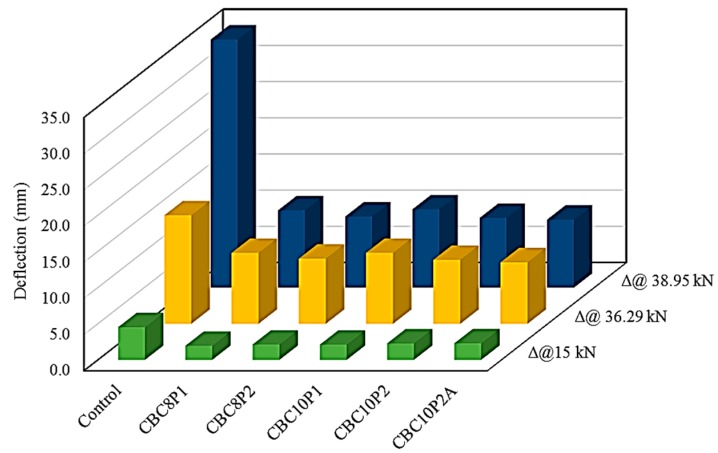
Deflection reduction of CEBNSM-strengthened beams.

Figure 5 shows the deflection reduction of the CEBNSM-strengthened beams at 15, 36.29, and 38.95 kN load with respect to the control beam. The last two loads were the yield and ultimate loads of the control beam. A15 kN load was added for better comparison, which was the first crack load for most of the strengthened beams. The aforementioned figure clearly showed that the deflection was reduced at these load levels. However, at 38.95 kN load, the difference was more pronounced than that of the other load levels. At the ultimate load stage, the deflection reduction was 69%, 71%, 68%, 72,% and 73% for the CBC8P1-, CBC8P2-, CBC10P1-, CBC10P2-, and CBC10P2A-strengthened beams, respectively.

### 3.3. Failure Modes

**Figure 6:**
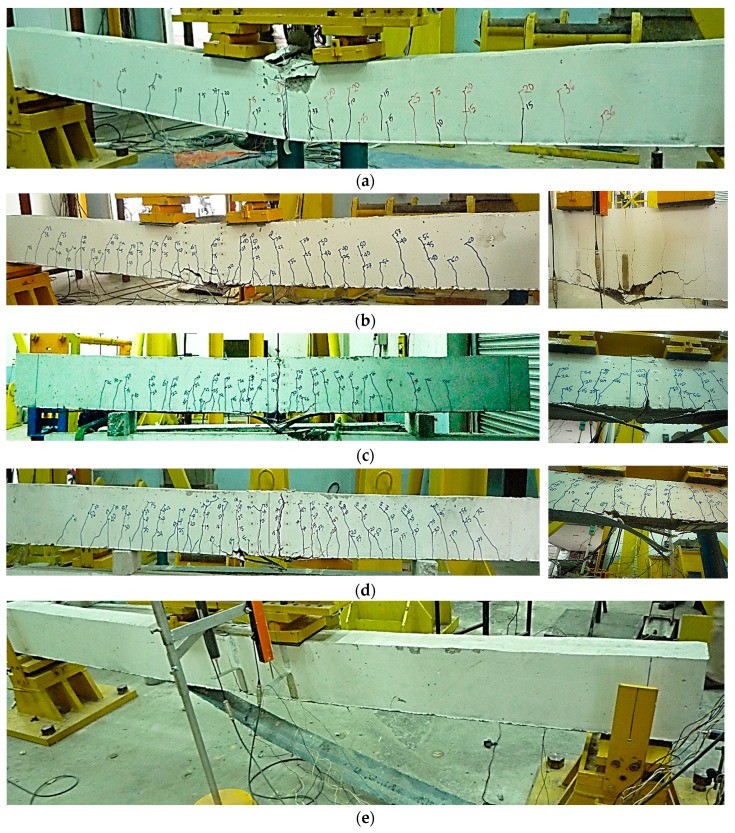

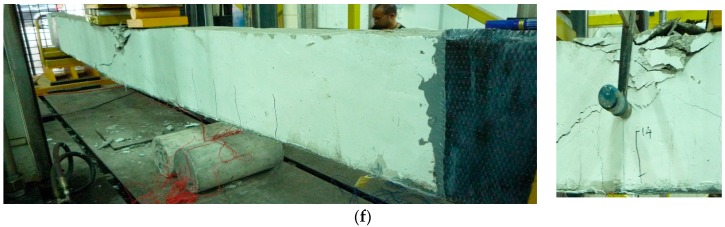
Failure modes of the control beam and the strengthened RC beams with close-up pictures at failure locations. (**a**) Control beam; (**b**) CBC8P1 beam; (**c**) CBC8P2 beam; (**d**) CBC10P1 beam; (**e**) CBC10P2 beam; (**f**) CBC10P2A beam.

Figure 6 illustrates the failure modes of the control beam (a) and the CEBNSM-strengthened beams (b–f). Except for the CBC10P2-strengthened beam, all of the CEBNSM-strengthened beams without anchorage showed EBR CFRP fabric fracture at the bottom tensile mid-span area, which was a manifestation of flexural failure modes. The 8 and 10 mm-diameter CFRP bars inside the NSM groove with a single layer of CFRP fabric showed a CFRP fracture at the middle, except for the CBC10P2-strengthened beam, which demonstrated debonding failure.

The CBC10P2-strengthened beam exhibited the premature debonding failure. After the yielding of the internal reinforcement, a cracking noise was detected like those detected from other CEBNSM-strengthened beams. At this stage, the CFRP fabric was stretched under high tensile strain, which was the maximum at the mid-span. Numerous new micro-cracks developed at the interface of the fabric and concrete cover, which expanded. At the maximum moment zone, the primary flexural crack widened and, at some point, the CFRP fabric could not maintain its curvature with the beam. Afterwards, the fabric lost its compatibility with the concrete surface and, with a bursting sound, the debonding initiated. The failure process was quick, and no sign of NSM failure was observed. Afterwards, the load was resisted only by the NSM reinforcement, which maintained an almost invariable load increment with increasing deflection. A concrete crushing failure was marked at this stage, and the machine was stopped.

Another beam was assessed with the same strengthening arrangement as the CBC10P2-strengthened beam, with two layers of U-CFRP wrap at the CFRP fabric cut-off location. Afterwards, the beam was called CBC10P2A. The beam survived against the debonding failure and showed the concrete crushing failure after steel bar yielding.

It can be seen that the CFRP fabric has a large influence on the failure mode experienced by CEBNSM-strengthened beams. The CFRP fabric was placed at the beam soffit; thus, it experienced the maximum tensile stress at the centre point of the mid-span. The NSM reinforcement was covered with concrete and was placed at least 8 mm away from the extreme bottom fibre. Consequently, the CFRP fabric played a dominant role in contributing to the ultimate flexural capacity as well as the failure modes. When maximum tensile strain in fabric reached closer to the rupture strain of the CFRP before attaining the maximum strain in concrete, the beam failed due to rupture of the CFRP fabric. Before that, the reinforcing steel in the tension area reached the plastic range.

### 3.4. Cracking Behaviour

During the test, cracking was clearly visualised into two different stages, which were the crack formation phase and the crack stabilisation phase. A digital crack microscope (DinoLite) was used to measure cracks at the steel bar level within the maximum moment region and was stored in a laptop. The cracks were documented after the appearance of the first crack and the subsequent crack formation at different load levels. Depending on the strengthening scheme and bond properties of concrete and internal steel, various crack spacing and widths were monitored for different beams. After the crack stabilisation period, new crack formations were stopped, whereas existing cracks were widened to maintain the same crack spacing.

#### 3.4.1. Crack Spacing

Crack spacing is a major parameter associated with crack width and deflection. Crack spacing is influenced by the concrete cover, strengthening scheme, internal bar spacing, bond properties, and strain distribution of different internal structural components.

According to the strain compatibility, the minimum crack (*s*_r0_) spacing can be expressed as the nearest point to a present crack at which a fresh crack can develop, where the concrete again reaches the tensile strength (Equation (1)). It can be expressed as

(1) $$s_{r0}=\frac{f_{\text{ctm}}\emptyset_{s}}{4\tau_{\text{bm}}\rho_{\text{ef}}}=\left(\frac{f_{\text{ctm}}A_{c,\text{eff}}}{\tau_{\text{bm}}\sum u}\right)$$

where $f_{\text{ctm}}$ = mean tensile strength of concrete; $\emptyset_{s}$ = nominal diameter of reinforcement; $\tau_{\text{bm}}$ = average bond stress along the disturbed zone; $\rho_{\text{ef}}$ = effective reinforcement ratio; $A_{c,\text{eff}}$ = effective concrete area in tension; and ∑u = (sum of) perimeter(s) of reinforcing bar(s).

According to [38,39], crack spacings were supposed to fluctuate between *s*_r.min_ = *s*_r0_ and *s*_r,max_ = 2*s*_r0_. Various researchers proposed different values of average (mean) crack spacing, which varied from 1.33 to 1.54 times the minimum value (Equations (2) and (3)), whilst maximum crack spacing can be expressed as *s*_r,max_ = 2*s*_r,min_.

(2) $$\frac{s_{r,\text{min}}}{s_{r,\text{mean}}}=0.67\;to\;0.77$$

(3) $$\frac{s_{r,\text{max}}}{s_{r,\text{mean}}}=1.33\;to\;1.54$$

The minimum, mean, and maximum crack spacings were determined based on the recorded data shown in Table 4. The maximum and mean crack spacings of CEBNSM-strengthened beams were comparatively lower than that of the control beam, although the number of cracks was greater. This information affirmed the better energy dissipation in the CEBNSM-strengthened beams.

Table 4 shows the maximum, minimum, and average crack spacings, along with the number of cracks that appeared on the tested beams. The minimum, maximum, and mean crack spacings of the strengthened beams were observed to be 45, 110, and 69 mm, respectively. The average crack spacing of CEBNSM-strengthened beams maintained a range between 64 and 77 mm, whereas the average crack spacing of the control beam was 109 mm. The number of cracks that appeared on the strengthened beam was almost the same, and its average was approximately 35, compared with 21 cracks on the control beam. The CBC8P1-strengthened beam exhibited the highest number of cracks (39 cracks), whereas the CBC8P2-strengthened beam showed the minimum number of cracks (31 cracks). The strengthened beams displayed many cracks with small width, whereas the unstrengthened beam had fewer cracks with large width. Owing to beam deformation due to the applied loads, the strengthening material in strengthened beams creates a tensile force that equalises the internal bending forces so that less deformation occurs compared to the unstrengthened beam [40].

**Figure 7:**
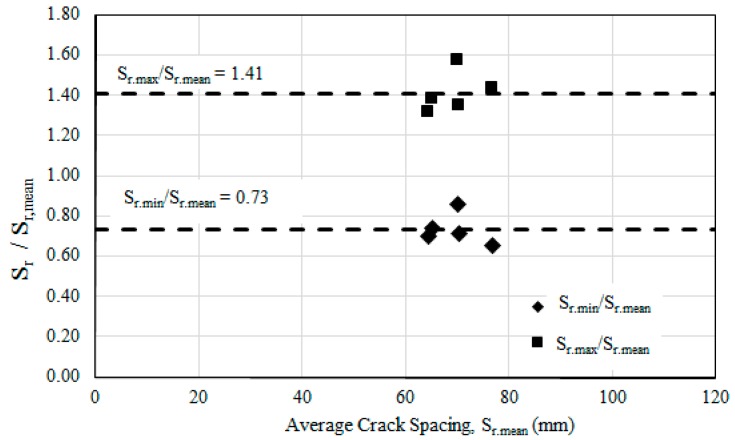
Relationship of maximum and minimum crack spacing against mean crack spacing.

Figure 7 shows the ratios of minimum-to-average and maximum-to-average crack spacings of the CEBNSM-strengthened RC beams. The experimental result shown in Figure 7 reveals the average maximum and minimum crack spacing ratio as 1.41 *S*_r.max_ and 0.73 *S*_r.min_, which complies with the limit suggested in Equations (2) and (3). Moreover, the ratio of the average *S*_r.max_ and *S*_r.min_ was 1.94, which was close to the findings of Borosnyói [39].

#### 3.4.2. Crack Width

**Figure 8:**
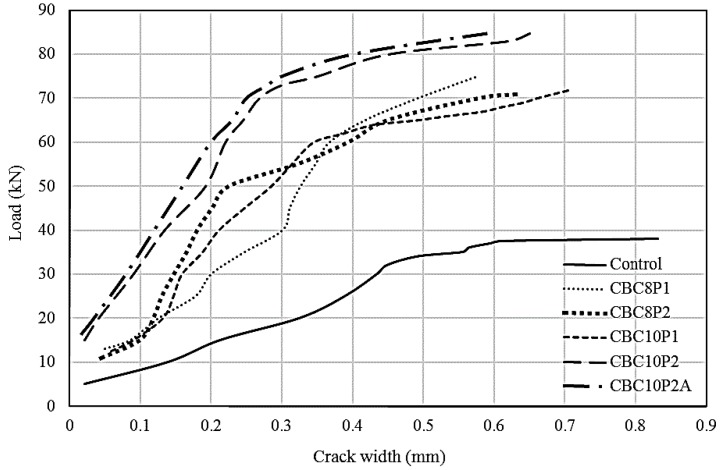
Crack widths of CEBNSM-strengthened beams against incremental load.

The flexural crack width of beams was measured across the main reinforcement position in the maximum moment region at different load levels with the help of a crack-measuring microscope. For all of the beams, the cracks were measured beyond their yield load-carrying limits, which were close to their failure stage. The minimum-to-maximum range of the first crack improvement was 118% to 230% compared with the control beam. Figure 8 shows the trend of the crack width of strengthened RC beams compared with the control beam. For all cases, the strengthened beams exhibited less crack width and higher first crack load compared with the control beam. It is possible to characterize the trend of crack width into three groups, where the control beam exhibited the widest crack width. The CBC8P1-, CBC8P2-, and CBC10P1-strengthened beams showed moderate decrements in crack width, whereas the CBC10P2- and CBC10P2A-strengthened beams demonstrated the stiffest response in widening crack width compared with the control beam. Up to the yielding stage, the formation of crack width was stiffer, which widened faster beyond the region as the stiffness of the beam decreased.

If a single load is considered, then comparing the crack width of different beams would be easier. As a 35 kN load was close to the yield load of the control beam, comparing the crack width with this value would be easier. At a 35 kN load, a 0.56 mm crack width was developed in the control beam. The corresponding crack widths formed at this load were 0.17, 0.25, 0.19, 0.11, and 0.10 mm for the CBC8P1-, CBC8P2-, CBC10P1-, CBC10P2-, and CBC10P2A-strengthened beams, respectively.

The ACI-318 code included provisions for cracking control based on crack width limits of 0.4 and 0.33 mm for interior and exterior applications, respectively. A permissible crack width of between 0.4 and 0.53 mm was selected by Frosch [41]. A service load steel stress of 0.6 Fy was assumed, and simplified design curves were generated based on this assumption. Barris [38] selected and analysed the experimental FRP RC beams with a crack width of between 0.5 and 0.7 mm. Among the several code requirements, 0.33 mm [42] was the most conservative value. For comparison purposes, the load corresponding to this crack width (listed in Table 5) will be determined. The service load (60% of the ultimate load) and its corresponding crack width are presented in Table 5. Although the service load of strengthened beams was higher, their corresponding service crack width was less than that of the control beam. The average load of the strengthened beams at the 0.33 mm crack width was 63.6 kN. This mean value represented a load corresponding to an average of 75% of their corresponding ultimate load-carrying capacity.

### 3.5. Stiffness Assessment

Stiffness is one of the dominant characteristics of RC structures, given that the change of its value with the applied load influences the deflection and curvature of any structure. Stiffness depends significantly on the cracking, the load level, and the thickness of bonded material and adhesive. Stiffness can be characterised as the product of the modulus of elasticity and moment of inertia of a certain section. Bending stiffness is easily defined for a true homogenous material, such as steel. However, for RC, estimating bending stiffness is difficult, as it is controlled by cracking, creep, shrinkage, and load history. In the RC section, the moment of inertia is continuously changing, which is termed as the effective moment of inertia (*I*_eff_) after exceeding the cracking moment (*M*_cr_) instead of using the gross moment of inertia (*I*_g_). For the full crack formation of the beam, *I*_eff_ should be referred to as the cracked moment of inertia (*I*_cr_) of the cracked transformed section. With the formation of flexural cracks, the neutral axis also keeps changing its position, which is also a significant challenge for the appropriate estimation of bending stiffness.

The RC beam section significantly varies with the un-cracked and cracked stages because of the applied load. Bending stiffness can be estimated from the displacement data coming from the LVDTs placed along the beam length (Figure 1). By using elastic bending theory in the displacement-based equation, calculating the experimental bending stiffness using Equation (4) is possible [43].

(4) $${\left(EI\right)}_{\text{exp}}=\frac{Pa\left(3l^{2}-4a^{2}\right)}{48\delta_{\text{exp}}}$$

Here, *P*, *l*, *a*, and δ_exp_ represent the applied service load, clear span of the RC beam, shear span of the beam, and the maximum mid-span experimental deflection at service load, respectively.

Another approach for the determination of bending stiffness is to evaluate the curvature of the beam at bending due to the applied experimental load. For that purpose, the moment–curvature relationship of the RC beam should be developed. Three approaches are employed to establish this relationship, as follows: (a) analyse the strain of the top compression fibre and bottom steel; (b) analyse the strain of the bottom and top steel; and (c) analyse the strain of the top fibre and CFRP bar.

(5) $${\left(EI\right)}_{\text{exp}}=\frac{M}{\varphi }$$

(6) $$\varphi =\frac{\varepsilon_{c}+\varepsilon_{s}}{d}$$

**Figure 9:**
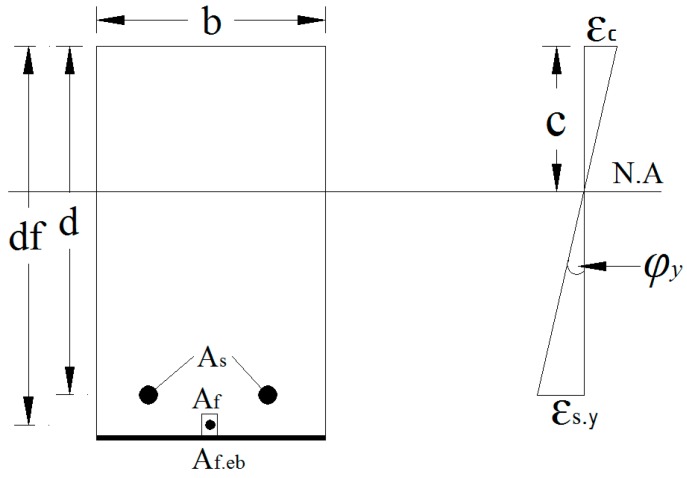
Cross-sectional view of the strengthened beamshowing neutral axis (NS) location and curvature. *b* is the width of the beam, *d* is the effective depth of the beam, *d*f is the effective depth of the NSM reinforcement, *A*_s_ is the area of the main reinforcement, *A*_f_ is the area of the NSM reinforcement, *A*_f__.__eb_ is the area of the externally bonded reinforcement, N.A is the neutral axis of the section, *c* is the depth of the neutral axis, ε_c_ is the compressive strain of the concrete, ε_s.y_ is the tensile strain of the main reinforcement and φ_y_ is the curvature of the section.

For the analysis, curvature and neutral axis location was determined by using the tensile and compression strain values of steel and concrete from their respective strain gauges [44]. From Figure 9 and Equations (5) and (6), the bending stiffness of the beams can be calculated. A moment–curvature relationship was developed using the extreme tension–strain values from the strain gauge of the internal steel bar and the extreme compression strain gauge values from the top of the mid-span.

**Figure 10:**
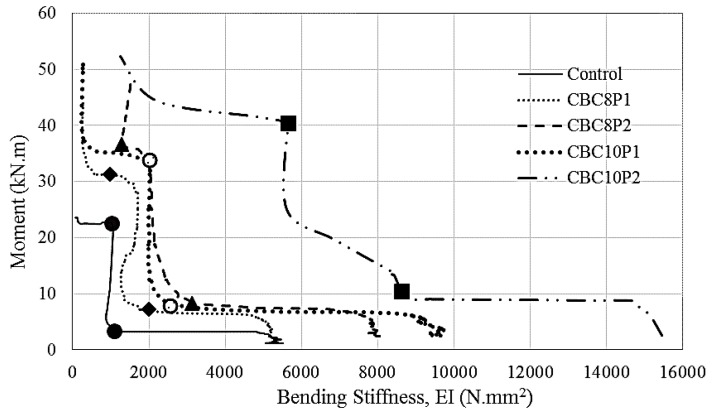
Bending stiffness of the strengthened beams.

Figure 10 depicts the moment versus bending stiffness diagram, where the first crack and the yield of the beams were marked in the diagram. The strain gauge of CBC10P2A-strengthened beams yielded erroneous data, which were excluded from the analysis. The overall shape of the moment versus bending stiffness curve was formed like an “L”. The stiffness was initially high and then constantly decreased until the appearance of the first crack in the beam. Afterwards, the moment increased with almost an invariable amount of stiffness until the yielding of the beam. The stiffness again decreased with a minute change of moment increment. Then, the moment increased again with an insignificant change in stiffness.

For all cases, as expected, the CEBNSM-strengthened beams exhibited a superior moment–stiffness relationship compared with the control beam. The bending stiffness was initially high, as expected, because of the un-cracked stage of the beam section. The initial stiffness of the control beam was 5512 N·mm^2^. The initial stiffness values of the strengthened beams were 5203, 8042, 9653, and 15,453 N mm^2^ for the CBC8P1-, CBC8P2-, CBC10P1-, and CBC10P2-strengthened beams, respectively.

With the increase in load application, the stiffness decreased and formed the knee of the “L”, where the first crack appeared. This first crack stiffness of the control beam was 1,105 N·mm^2^. The intermediate stiffness at the first crack of the CEBNSM-strengthened beams was 2008, 3142, 2553, and 8637 N·mm^2^ for the CBC8P1-, CBC8P2-, CBC10P1-, and CBC10P2-strengthened beams, respectively. No noticeable difference in stiffness was observed after the first crack, and a radical realignment was visualised for this curve. An almost straight vertical line was formed where the moment increased with a steady rate. The CBC10P2-strengthened beam showed a gradually-decreasing stiffness with the increase in the moment capacity from the crack moment to the yield moment.

At the yield moment, the stiffness of the control beam was 1032 N·mm^2^. The stiffness at the yield moment of the CBC series beams was 983, 1292, 2002, and 5656 N·mm^2^ for the CBC8P1-, CBC8P2-, CBC10P1-, and CBC10P2-strengthened beams. After crossing the yield moment point, the beams showed an almost constant decrease in stiffness with a negligible moment increment. Then, the moment capacity increased again without any appreciable change in stiffness up to failure.

## 4. Simulation Method and Verification

### 4.1. Moment-Rotation Approach

The behaviour of RC beams is commonly simulated using the moment-curvature approach. The moment-curvature approach is usually capable of simulating strengthened RC beams with reasonable accuracy and with less computation needed compared to finite element modelling. However, the moment-curvature models are generally analytical models that rely heavily on empirical formulations derived specifically for the type of strengthening used on the RC beam. In order to use the moment-curvature approach for new strengthening methods such as the hybrid strengthening proposed in this paper, some recalibrations or modifications of these empirical formulations would be necessary. Yet, due to the small number of samples tested in this paper, the resulting empirical formulations may not be as accurate as needed. As such, an alternative method was used—the moment-rotation approach [45,46,47]. The moment-rotation approach requires no calibrations, as it is able to directly simulate the mechanisms of the RC beam—such as tension stiffening, crack formation, and crack widening—without the need for empirical formulations that are normally needed to indirectly simulate these mechanisms.

#### 4.1.1. Tension Stiffening Analysis

**Figure 11:**
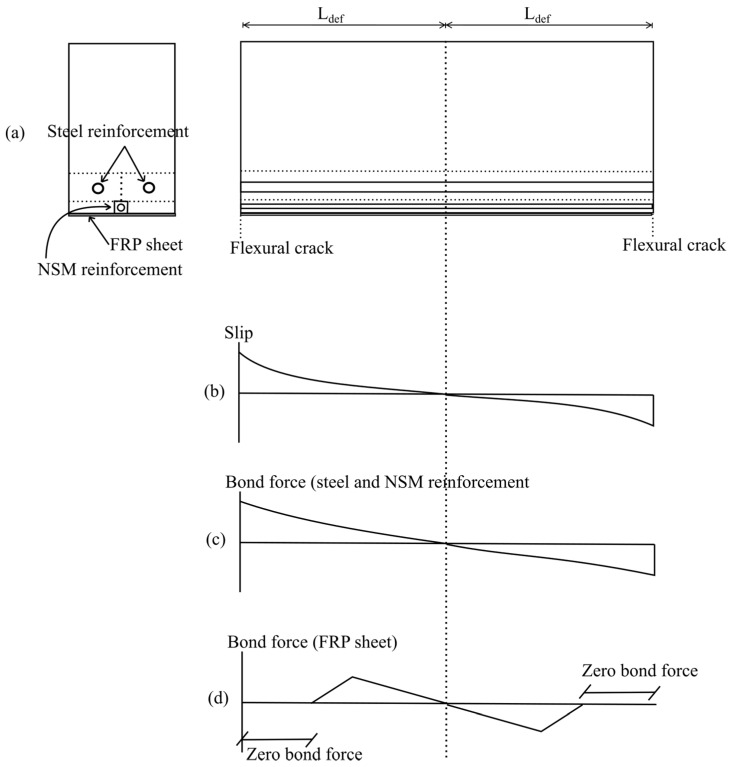
Tension stiffening analysis. FRP: fibre reinforced polymer; NSM: near surface mounted.

The moment-rotation approach uses the partial interaction theory to simulate the slip of reinforcements in RC beams, thus allowing the tension stiffening to be directly simulated. In this paper, the segmental method as presented by Visintin, et al. [47] will be used. Consider Figure 11a, which shows a beam segment of length 2*L*_def_ located between two flexural cracks. The slip of reinforcements would be at maximum at the location of the flexural cracks. As the bond stress acting on the reinforcements reacts against the slip of the reinforcements, the slip of the reinforcements would be gradually reduced, as the force acting on the reinforcements would be transferred to the adjacent concrete. Due to symmetry of forces, the slip of reinforcements would tend to zero at the middle of the beam segment, as shown in Figure 11b. As such, the analysis area can be reduced to length *L*_def_.

A numerical method similar to what was used by a previous researcher [48] was used to simulate the slip of steel and NSM reinforcements. The beam segment is discretized into small elements of size with length of each element (*L*_s_) taken as 0.1 mm, where the stress and strain acting in each element is assumed to be constant due to its small size. The maximum element for the analysis, *i*_max_ = *L*_s_*L*_def_. The steel reinforcement is assumed to slip by a certain amount, and the load needed to cause this slip is assumed. The load and slip values for each element are then solved numerically and the load is adjusted until the slip is reduced to zero at the middle of the beam segment. The process is repeated until a load–slip relationship is obtained. The bond–slip model by CEB-FIP [49] was used to determine the bond force acting on the steel reinforcement.

The method used to simulate the slip of NSM reinforcement is similar to the method used by Shukri, et al. [46]. The numerical procedure of the NSM reinforcement is nearly the identical to the steel reinforcement, except the bond–slip model by De Lorenzis, et al. [50] was used to determine the bond force of the NSM reinforcement:

(7) $$\tau =\tau_{\text{max}-n}{\left(\frac{\delta }{\delta_{\text{max}-n}}\right)}^{\alpha }\;for\;\delta \leq \delta_{\text{max}-n}$$

(8) $$\tau =\tau_{\text{max}-n}{\left(\frac{\delta }{\delta_{\text{max}-n}}\right)}^{\alpha^{\prime }}\;for\;\delta >\delta_{\text{max}-n}$$

where τ is the bond stress, τ_max−n_ is the maximum bond stress, δ is the slip, and δ_max−n_ is slip corresponding to τ_max−n_. The full list of parameters used for the bond–slip model for NSM FRP bars is provided in Table 6, where the parameters are empirically derived by De Lorenzis [50] for RC beams strengthened with NSM FRP ribbed bars, with the exception of τ_max−n_, which was 21 MPa based on the value of bond strength given by the manufacturer of the Sikadur^®^ 30 epoxy adhesive.

A numerical tension stiffening analysis was also applied to the FRP sheet. The bilinear bond-slip model by Lu, et al. [51] was used:

(9) $$\tau =\tau_{\text{max}-s}\sqrt{\left(\frac{\delta }{\delta_{o}}\right)}\;\text{for}\;\delta \leq \delta_{o}$$

(10) $$\tau =\tau_{\text{max}-s}\left(\frac{\delta_{f}-\delta }{\delta_{f}-\delta_{o}}\right)\;\text{for}\;\delta_{o}<\delta \ll \delta_{f}$$

(11) $$\tau =0\;\text{for}\;\delta >\delta_{f}$$

where,

(12) $$B_{w}=\sqrt{\frac{2.25-b_{f}/b_{c}}{1.25+b_{f}/b_{c}}}$$

(13) $$\tau_{\text{max}-s}=1.5B_{w}f_{t}$$

(14) $$\delta_{o}=0.0195B_{w}f_{t}$$

(15) $$\delta_{f}=2G_{f}/\tau_{\text{max}}$$

(16) $$G_{t}=0.308B_{w}^{2}\sqrt{f_{t}}$$

Unlike the steel and NSM reinforcement, the bond between the FRP sheet and the concrete can be reduced to zero if the slip is larger than the maximum slip as determined using the bond–slip model. As more slip occurs, nearly all the bond along the beam segment will be reduced to zero. This causes the bond force for the FRP sheet to vary significantly from the bond force for steel and NSM reinforcements, as shown in Figure 11c,d. Importantly, the small amount of bond for the FRP sheet in this state causes the tension stiffening contribution of the FRP sheet to the RC beam to be very insignificant compared to the tension stiffening contribution of steel and NSM reinforcement.

A bilinear stress–strain relationship for steel reinforcement was used. For the FRP bar and sheet, a linear stress–strain relationship was used.

#### 4.1.2. Moment-Rotation Analysis

**Figure 12:**
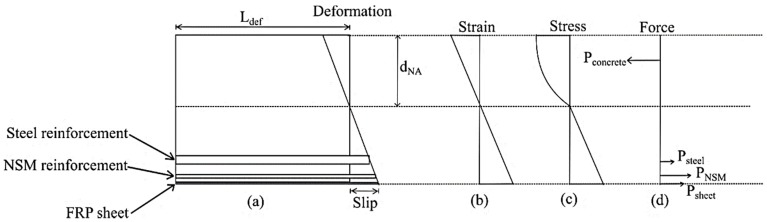
Moment-rotation analysis.

The procedure for the moment-rotation approach prior to the occurrence of flexural cracking is similar to the moment-curvature approach. As moment M is applied, it causes a rotation θ to occur on the beam segment. A deformation profile (as shown in Figure 12a) is thus formed due to this rotation. To account for the formation of concrete wedges and the occurrence of concrete crushing, the size-dependent stress–strain relationship proposed by Chen, et al. [52] was used, which allows the concrete stress–strain relationship to be adjusted to length *L*_def_. The concrete stress–strain model by Popovics [53] was used as the base model that was adjusted for concrete size:

(17) $$\sigma_{c}=f_{c}\left(\frac{\left(\frac{\varepsilon_{c}}{\varepsilon_{a}}\right)r}{r-1+{\left(\frac{\varepsilon_{c}}{\varepsilon_{a}}\right)}^{r}}\right)$$

where σ_*c*_ is the concrete stress, *f*_c_ is the concrete strength, ε_c_ is the concrete strain. The parameters *r* and peak strain, ε_a_ are determined as:

(18) $$r=\frac{E_{c}}{E_{c}-f_{c}/\varepsilon_{a}}$$

(19) $$\varepsilon_{a}=4.76\times 10^{-6}\left(f_{c}\right)+2.13$$

where *E*_c_ is the elastic modulus of concrete. It should be noted that Equation (19) was proposed by Chen, et al. [52], based on their research. To obtain the adjusted stress–strain relationship of concrete, σ_c_/ε_c-sd_—where ε_c-sd_ is the size adjusted strain—the size dependent strain for concrete is then determined as:

(20) $$\varepsilon_{c-\text{sd}}=\left(\varepsilon_{c}-\sigma_{c}/E_{c}\right)\left(\frac{100}{L_{\text{def}}}\right)+\varepsilon_{c}$$

While the beam is in a state with no flexural crack, the forces acting on the reinforcements can be determined using the strain and stress profile in Figure 12b,c. The depth of neutral axis, d_NA_ is then adjusted until an equilibrium of forces is achieved and the value of moment M is then determined from forces in Figure 12d.

Further rotation will cause a larger deformation, and once the strain in the tensile region reaches the concrete cracking strain, a flexural crack is assumed to have appeared on the beam segment. The force acting on the steel reinforcement, NSM reinforcement, and FRP sheet is determined using the deformation profile in Figure 12a, and the load–slip relationships determined using the tension stiffening analysis. The neutral axis is then adjusted to achieve equilibrium of forces, and the moment M is determined. The process is repeated to obtain a moment-rotation relationship. To obtain a moment-curvature relationship, then, is just a matter of dividing the rotation by *L*_def_. The load–deflection of the beam can then be determined from the moment-curvature relationship using the double integration method.

**Figure 13:**
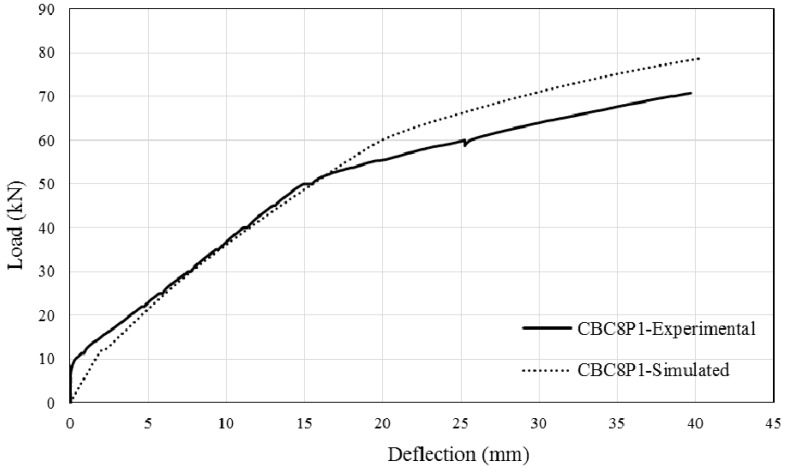
Experimental and Simulated output of CBC8P1.

**Figure 14:**
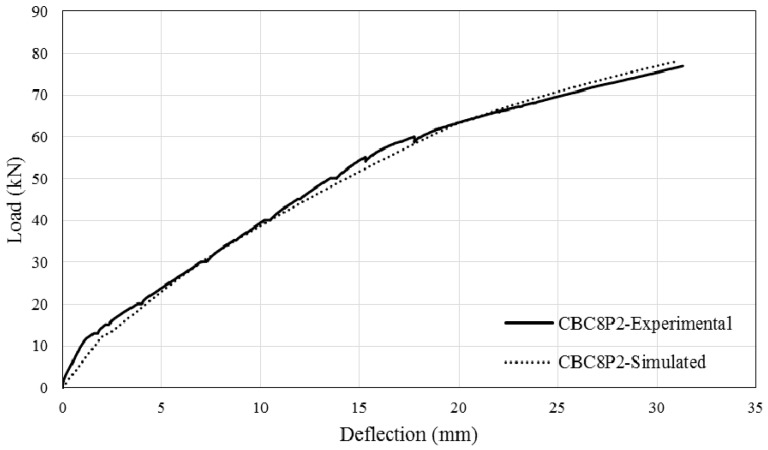
Experimental and Simulated output of CBC8P2.

**Figure 15:**
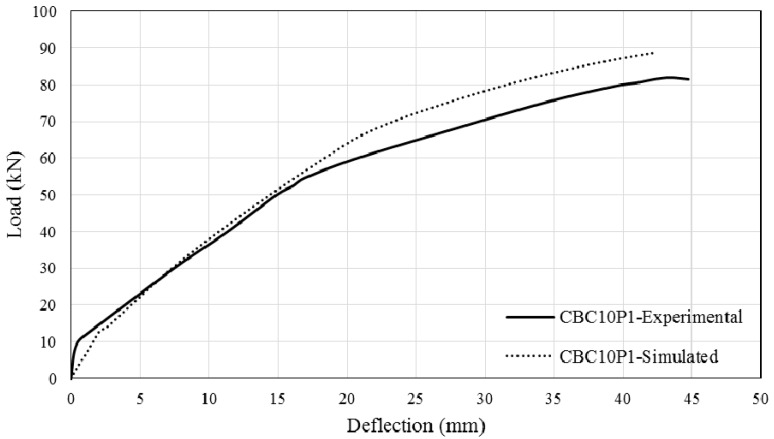
Experimental and Simulated output of CBC10P1.

**Figure 16:**
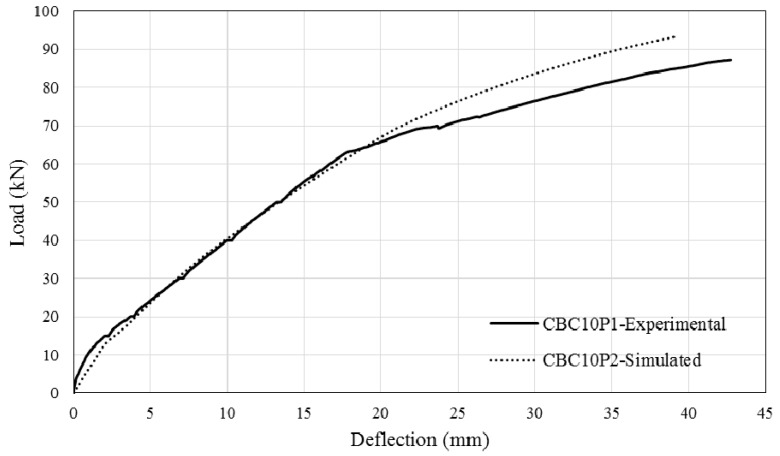
Experimental and Simulated output of CBC10P2.

The comparison between simulated and experimental load–deflection curves are as shown in Figure 13, Figure 14, Figure 15 and Figure 16. It can be seen that the simulated curve follows the general shape of the experimental curves reasonably well; the tension stiffening analysis was able to simulate the beam behaviour with considerable accuracy. However, the method is currently unable to simulate the concrete cover separation failure of CEBNSM-strengthened beams with good accuracy. Further work is needed into this area.

## 5. Conclusions

This study introduces the CEBNSM method for strengthening RC beams, which involves strengthening beams using NSM CFRP round bar in combination with EBR CFRP fabric at the beam soffit. The effect of the variable NSM bar diameter, the thickness of the EBR CFRP fabric, and the anchorage performance was evaluated based on a four-point bending experimental test. A simulation method based on the moment-rotation approach was used to predict the deflection of the CEBNSM-strengthened RC beams. The following summary can be drawn from the experimental and analytical outcomes.

- The first crack, yield, and ultimate load of the CEBNSM-strengthened beams significantly increased compared with the control beam. The increment of the first crack load was the highest (230%) among the three load levels, which is particularly important for serviceability performance. The maximum ultimate load-carrying capacity increased to 170% over that of the control beam.
- A trilinear load–deflection response was detected, whereas a considerable reduction of the deflection for all of the strengthened beams was witnessed at the ultimate stage. The stiffness of the strengthened beam significantly increased at all levels of load compared with that of the control beam.
- All of the strengthened beams exhibited flexural failure, except for the CBC10P2-strengthened beam, which was strengthened using a double-ply CFRP fabric with a 10 mm-diameter NSM CFRP bar. However, this debonding failure was successfully eliminated by using CFRP U-Wrap anchorage at the fabric curtailment location.
- The average crack spacing of the strengthened beams was 64 to 77 mm, which was smaller than that of the control beam (109 mm). The number of cracks was also more significant (average of 35 cracks) than that of the control beam (21 cracks), which affirmed the enhanced energy dissipation of the strengthened beams. Furthermore, the crack width of the strengthened beams was significantly reduced.
- The strain value of steel and concrete for the strengthened beams was less than that of the control beam. The strain values of the NSM bar and the EBR fabric showed the perfect distribution of the strain by strengthening reinforcement after the yielding of the internal steel bar.
- The moment-rotation approach was applied to simulate the behaviour of CEBNSM-strengthened RC beams and was able to give good accuracy.

## Figures and Tables

**Table 1:** Test matrix of the experimental program.

Serial. No.	Notation	Description	Strengthening details
1	CB	Control RC beam	Without strengthening
2	CBC8P1	8 mm φ NSM CFRP bar and 1 ply of EBR CFRP fabric	CFRP bar: 1–8 mm φ (*L* = 2900 mm) CFRP fabric: 2900 × 125 × 0.17 mm^3^
3	CBC8P2	8 mm φ NSM CFRP bar and 2 ply of EBR CFRP fabric	CFRP bar: 1–8 mm φ (*L* = 2900 mm) CFRP 1st fabric: 2900 × 125 × 0.17 mm^3^ CFRP 2nd fabric: 2600 × 125 × 0.17 mm^3^
4	CBC10P1	10 mm φ NSM CFRP bar and 1 ply of EBR CFRP fabric	CFRP bar: 1–10 mm φ (*L* = 2900 mm) CFRP fabric: 2900 × 125 × 0.17 mm^3^
5	CBC10P2	10 mm φ NSM CFRP bar and 2 ply of EBR CFRP fabric	CFRP bar: 1–10 mm φ (*L* = 2900 mm) CFRP 1st fabric: 2900 × 125 × 0.17 mm^3^ CFRP 2nd fabric: 2600 × 125 × 0.17 mm^3^
6	CBC10P2A	NSM CFRP bar, EB 2 ply CFRP fabric and 2 ply U-wrap end anchorage	CFRP bar: 1–10 mm φ (2900 mm) CFRP fabric: 2900 × 125 × 0.34 mm^3^ CFRP U-wrap anchorage: 2 ply (625 × 125 × 0.34 mm^3^)

**Table 2:** Material properties of strengthened specimens.

Material	Mechanical property	Result
Concrete	Compressive strength (MPa)	50.1
	Flexure strength (MPa)	5.5
	Elastic modulus (GPa)	33.26
Steel 12 mm φ (Internal bottom reinforcement)	Yield stress (MPa)	529
	Ultimate strength (MPa)	587
	Elastic modulus (GPa)	200
	Elongation (%)	21
Steel 10 mm φ (Internal top reinforcement)	Yield stress (MPa)	521
	Ultimate strength (MPa)	578
	Elastic modulus (GPa)	200
	Elongation (%)	20
Steel 8 mm φ (Internal shear reinforcement)	Yield stress (MPa)	380
	Ultimate strength (MPa)	450
	Elastic modulus (GPa)	200
	Elongation (%)	29
CFRP bar-12 mm φ	Ultimate strength (MPa)	2,400
	Elastic modulus (GPa)	165
	Ultimate strain (%)	1.6
CFRP Fabric (SikaWrap-301C) [35]	Ultimate strength (MPa)	4,900
	Elastic modulus (GPa)	230
	Ultimate strain (%)	2.1
Epoxy (Sikadur^®^) 30 [36]	Compressive strength	70–80 MPa (15 °C); 85–95 MPa (35 °C)
	Tensile strength	14–17 MPa (15 °C); 16–19 MPa (35 °C)
	Shear strength	24–27 MPa (15 °C); 26–31 MPa (35 °C)
Epoxy (Sikadur^®^) 330 [37]	Tensile strength (MPa)	30
	Elastic modulus–Flexural (MPa)	3,800
	Elastic modulus–Tensile (MPa)	4,500

**Table 3:** Summary of the experimental test results.

Beam ID	*P*_cr_ (kN)	Δ_cr_ (mm)	*P*_y_ (kN)	Δ_y_ (mm)	*P*_u_ (kN)	Δ_u_ (mm)	Failure modes
CB	5	0.5	36	15.0	39	34.3	FFC
CBC8P1	11	1.5	50	14.9	71	39.7	FFF
CBC8P2	13	1.9	55	15.2	77	31.3	FFF
CBC10P1	13	1.6	54	16.6	82	43.3	FFF
CBC10P2	15	2.3	69	23.7	87	42.7	CFD
CBC10P2A	16	2.8	80	24.7	105	47.9	FFC

*P*_cr_ = first crack load; *P*_y_ = yield load; *P*_u_ = ultimate load; ∆_cr_ = deflection at 1st crack; ∆_y_ = deflection at yield of steel; ∆_u_ = mid-span deflection at failure load; FFC = flexural failure (concrete crushing after steel yielding); FFF = flexure failure due to FRP rupture; CFD = CFRP fabric delamination.

**Table 4:** Experimental crack spacing and analysis.

Beam No.	*S*_r.max_ (mm)	*S*_r.min_ (mm)	*S*_r.mean_ (mm)	No. cracks
CB	140	75	109	21
CBC8P1	85	45	64	39
CBC8P2	110	50	77	31
CBC10P1	95	50	70	38
CBC10P2	90	48	65	34
CBC10P2A	110	60	70	33

*S*_r.max_ denotes the maximum crack spacing, *S*_r.min_ denotes the minimum crack spacing and S_r.mean_ denotes the mean crack spacing.

**Table 5:** Equivalent experimental load at *w* = 0.33 mm.

Beam ID	*P*_cr_ (kN)	*P*_serv_ (kN)	*w*_serv_ (mm)	Load (kN) at *w* = 0.33 mm	% of Pu
Control	5.0	23.4	0.34	22	56
CBC8P1	10.9	42.5	0.18	56	79
CBC8P2	13.0	46.1	0.31	54	70
CBC10P1	12.6	49.0	0.28	58	71
CBC10P2	15.0	52.4	0.19	74	85
CBC10P2A	16.5	63.1	0.21	76	72

*P*_cr_ = 1st crack load, *P*_serv_ = Service load (60% of the ultimate load), *w*_serv_ = crack width at service load.

**Table 6:** Parameters for bond-slip of NSM FRP.

Parameter	Value
δ_max_ (mm)	0.319
τ_max−n_ (mm)	21
α	0.65
α’	−0.88
